# Modeling of wave run-up by applying integrated models of group method of data handling

**DOI:** 10.1038/s41598-022-12038-2

**Published:** 2022-05-18

**Authors:** Amin Mahdavi-Meymand, Mohammad Zounemat-Kermani, Wojciech Sulisz, Rodolfo Silva

**Affiliations:** 1 Institute of Hydro-Engineering, Polish Academy of Sciences, Warsaw, Poland; 2 Water Engineering Department, Shahid Bahonar University of Kerman, Kerman, Iran; 3 Instituto de Ingeniería, Universidad Nacional Autónoma de México, 04510 Mexico City, Mexico

**Keywords:** Civil engineering, Computational science, Computer science

## Abstract

Wave-induced inundation in coastal zones is a serious problem for residents. Accurate prediction of wave run-up height is a complex phenomenon in coastal engineering. In this study, several machine learning (ML) models are developed to simulate wave run-up height. The developed methods are based on optimization techniques employing the group method of data handling (GMDH). The invasive weed optimization (IWO), firefly algorithm (FA), teaching–learning-based optimization (TLBO), harmony search (HS), and differential evolution (DE) meta-heuristic optimization algorithms are embedded with the GMDH to yield better feasible optimization. Preliminary results indicate that the developed ML models are robust tools for modeling the wave run-up height. All ML models’ accuracies are higher than empirical relations. The obtained results show that employing heuristic methods enhances the accuracy of the standard GMDH model. As such, the FA, IWO, DE, TLBO, and HS improve the *RMSE* criterion of the standard GMDH by the rate of 47.5%, 44.7%, 24.1%, 41.1%, and 34.3%, respectively. The GMDH-FA and GMDH-IWO are recommended for applications in coastal engineering.

## Introduction

Wave-induced inundation in coastal zones is a very complex phenomenon of fundamental importance for many disciplines. The design of coastal areas and coastal zone management strongly depend on the storm and tide-induced inundation studies. Moreover, wave-induced inundation is of fundamental importance for the erosion processes, the maintenance of beaches, and the biological processes in surges zones. Proper understanding of inundation and the prediction of flooding is crucial for hydraulic and coastal engineering and the sustainable development of coastal areas.

**Figure 1:**
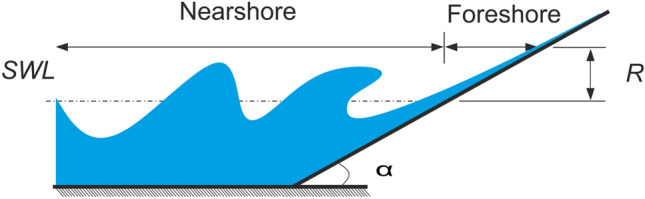
A schematic presentation of wave run-up on the foreshore.

Coastal areas are very vulnerable zones to storm and tide-induced inundation. Wave-induced inundation may cause severe damage in these areas and the consequences of severe flooding are often fatal for the coastal zone population, which constitutes from 10 to 20% of the global population. In fact, presently about 40% of the world's population lives within 100 km of the coast. One of the key elements describing storm and tide-induced inundation in coastal areas is a wave run-up height. Wave run-up is equal to the vertical distance of the water’s edge on a coastal structure or on the foreshore of the beach, which is varying with time. In Fig. 1, a schematic view of the run-up (*R*) process is shown.

In Fig. 1, *a* is the beach or structure slope, and *SWL* is the water level. Structure overtopping, dune erosion during storm conditions^1^, and sediment transport in the coastal zone^2^ are examples of some phenomena which the run-up processes may cause. In this respect, designing the protection structures in coastal areas cannot be made without considering the accurate prediction of the wave run-up parameters. Hence, the simulation of the general characteristics of the run-up is of great importance in coastal engineering.

In the past decades, numerous studies applied experimental tests^3–5^ and numerical modeling for simulating and predicting the wave run-up in coastal regions. In recent years modeling complex and nonlinear problems in science and engineering using machine learning (ML) models have received widespread attention and, in general, the capability of these approaches has been confirmed. Selected recent applications of ML in coastal engineering are discussed below.

Chang and Lin^6^ used an artificial neural network (ANN), harmonic model (HM), and global ocean tidal model (NAO.99b) to simulate tides at selected points. The comparisons of the results obtained by ANN with corresponding results obtained by applying HM and NAO.99b confirmed the superiority of the ANN model. Erdik and Savci^7^ simulated wave run-up on armored rock slopes by applying Takagi–Sugeno (TS) fuzzy models. The results showed that TS models provided better accuracy in simulating a run-up than the empirical methods of Van der Meer and Stam^8^. Shiri et al.^9^ used an adaptive neuro-fuzzy inference system (ANFIS), ANN, linear regression, and autoregressive methods for modeling sea-level variations. The results confirmed the superiority of ML techniques in comparison with the regression methods. Bonakdar and Etemad-Shahidi^10^ used the M5 model tree, Takagi–Sugeno fuzzy model, and empirical formulae to simulate wave run-up on rubble-mound structures. The study showed that the M5 results were better than the corresponding results obtained by the application of the remaining methods. Elbisy^11^ investigated the ability of multiple additive regression trees (MART) and ANN in the simulation of wave run-up. The results showed that MART is more accurate than ANN. Abolfathi et al.^12^ used the M5 decision tree algorithm for predicting wave run-up. The study showed that the M5 method provides more accurate results than a regression-based model. Pourzangbar et al.^13^ simulated non-breaking wave-induced scour depth at breakwaters with genetic programming and ANN methods. The study indicated that ML methods provide better results than empirical relations. Zhang et al.^14^ predicted real-time tidal levels using an integrative grey-GMDH neural network. The results revealed that the applied method can predict real-time tidal levels with high accuracy. In another study, Wang et al.^15^ predicted seawater levels by developing a hybrid model of ANFIS and wavelet decomposition. The results showed that the integrated method improved the accuracy of ANFIS. Gao et al.^16^ developed a multi-blocks fuzzy cognitive map model for predicting the steady turning motion of ships. The results confirmed the high performance of the proposed algorithm. Zeinali et al.^17^ applied two recurrent ANNs for the prediction of shoreline changes at Narrabeen, Australia. The results confirmed a good accuracy of the applied recurrent ANNs. Rehman et al.^18^ applied ANN and the response surface methodology (RSM) for wave run-up prediction. The obtained results showed that both the ANN and RSM are appropriate methods for the prediction of wave run-up. Also, the study of Yao et al.^19^ conformed a good accuracy of ANN in simulating wave run-up.

Recently, the applications of meta-heuristic algorithms integrated with ML methods have been reported in many studies. Masoumi Shahr-Babak et al.^20^ applied hybrid GMDH-HS to predict the uplift capacity of suction caisson in clay. They reported that the hybrid model can predict the suction caisson uplift capacity with acceptable accuracy. Najafzadeh et al.^21^ predicted bridge pier scour depth by developing several integrative GMDH methods. The study showed that particle swarm optimization (PSO) provides more accurate results than a genetic algorithm (GA) and gravitational search algorithm (GSA). Mahdavi-Meymand et al.^22^ used several meta-heuristic optimization algorithms to optimize ANFIS parameters to estimate the spillway aerator air demand in dams. They showed that hybrid models provide more accurate results than corresponding ML models. Dodangeh et al.^23^ applied GA and harmony search (HS) algorithm to optimize GMDH and support vector regression parameters for flood-susceptibility prediction. The results showed that both the GA and HS algorithms improved the accuracy of GMDH and SVR. Sharafati et al.^24^ confirmed the good accuracy of ANFIS-BBO in the prediction of long contraction scour depth. Sharafati et al.^25^ used teaching–learning-based optimization (TLBO), biogeography-based optimization (BBO), and invasive weed optimization (IWO) algorithms to optimize ANFIS parameters for the prediction of scour depth downstream of weirs. The results showed that ANFIS-IWO is a reliable technique for the prediction of scour depth.

Qaderi et al.^26^ developed a shuffled complex evolutionary (SCE) algorithm integrated with GMDH to predict bridge pier scour depth. The results showed a good performance of GMDH-SCE. Alizadeh et al.^27^ compared the performance of PSO, GA, and imperialist competitive algorithm (ICA) integrated with a support vector machine (SVM) for the estimation of drilling fluid density. The results showed the high performance of PSO in estimating drilling fluid density. Milan et al.^28^ applied three optimization algorithms, comprising particle swarm optimization (PSO), gray wolf optimization (GWO), and Harris hawk optimization (HHO) integrated with ANFIS for predicting optimal groundwater exploitation. The results indicated that all optimization algorithms increase the ANFIS accuracy. Haghbin et al.^29^ developed support vector regression (SVR) integrated with IWO for predicting channel sinuosity. The results showed that IWO significantly increases the accuracy of SVR.

In this study, invasive weed optimization (IWO), firefly algorithm (FA), teaching–learning-based optimization (TLBO), harmony search (HS), and differential evolution (DE) optimization techniques are embedded with GMDH to predict a two percent wave run-up height in coastal regions. It is worth noting that the applications of GMDH integrated with novel meta-heuristic models are scarce. Indeed, to the best knowledge of the authors, no study has ever developed and presented the application of hybrid models of GMDH-IWO for modeling problems in any field of research.

## Methods

In this section, a brief review of empirical relations for the prediction of wave run-up as well as the description of GMDH and optimization algorithms are presented.

### Empirical relations

In coastal engineering, several different wave run-up values can be investigated, such as the mean value (*R*), the 33 percent value of the wave run-up i.e. the significant run-up (*R*_*s*_), the two percent value of the wave run-up (*R*_*2%*_), etc. The mean run-up (*R*) can be calculated as the average run-up of all observed waves. However, the mean value is of limited interest for engineers and scientists. In this respect, other parameters might be used e.g. *R*_*s*_, *R*_*2%*_ and *R*_*10%*_. Note that *R*_*i%*_ refers to the run-up level reached and exceeded by *i*% of the incoming waves^30–36^. Numerous studies have been conducted on the regular wave run-up on smooth and rough beaches. Miche^31^ presented an equation to predict wave run-up for non-breaking waves:

1 $$\frac{{R_{\max } }}{H} = \sqrt {\frac{\pi }{2\alpha }}$$

where *R*_max_ is the maximum vertical run-up, *H* is the wave height, and *α* is the beach slope.

Hunt^37^ presented empirical relations to predict wave run-up on impermeable slopes based on breaking wave shape in the surf zone. The equation for standing waves on a steep slope was proposed in the following form:

2 $$\frac{{R_{\max } }}{H} \approx 3$$

where *R*_max_ may be calculated from:

3 $$R_{\max } = \frac{\tan \alpha }{{\sqrt {H/L_{0} } }} = \xi_{0}$$

in which $\xi_{0}$ is the surf similarity parameter or Iribarren number^38^, *L*_*0*_ is the deep-water wavelength

4 $$L_{0} = \frac{{gT^{2} }}{2\pi }$$

*g* is the gravitational acceleration and *T* is the wave period.

One of the first equations presented for estimating irregular wave run-up on mild uniform slopes (tanα ≤ 1/3) was proposed by Wassing^39^:

5 $$R_{u2\% } = 8H_{1/3} \tan \alpha$$

Ahrens^40^ studied irregular wave run-up on smooth-impermeable slopes (1/4 ≤ tan*α* ≤ 1.1) and suggested two equations for breaking and non-breaking waves. Coastal Engineering Manual^41^ analyzed Ahrens^40^ data and proposed two relations for predicting irregular wave run-up. Mase^42^ performed laboratory experiments on the irregular run-up on mild slopes (2° ≤ *α* ≤ 11.4°) and proposed an equation for estimating run-up due to breaking waves.

Van der Meer and Stam^8^ suggested the following relations for estimating wave run-up on smooth slopes for irregular waves:

6 $$\frac{{R_{u2\% } }}{{H_{s} }} = 1.5\gamma \xi_{\max }$$

where *H*_*s*_ is the significant wave height, *γ* is the reduction factor that depends on various parameters such as roughness, shallow water conditions, oblique wave attack, berms, and $\xi_{\max }$ is known as the Iribarren number corresponding to the maximum wave period.

In another study, Van der Meer and Stam^8^ applied the regression model proposed by Van der Meer^3^ and derived the following relations for predicting irregular wave run-up on permeable and impermeable slopes:

7 $$\begin{array}{*{20}l} {\frac{{R_{u2\% } }}{{H_{s} }} = 0.96\xi_{m} } \hfill & {1 \le \xi_{m} \le 1.5} \hfill \\ {\frac{{R_{u2\% } }}{{H_{s} }} = 1.17\xi_{m}^{0.46} } \hfill & {1.5 < \xi_{m} \le 3.1} \hfill \\ {\frac{{R_{u2\% } }}{{H_{s} }} = 1.97\xi_{m}^{0.46} } \hfill & {3.1 < \xi_{m} \le 7.5} \hfill \\ \end{array}$$

where $\xi_{m}$ is the Iribarren number corresponding to the average wave period.

Schimmels et al.^5^ proposed the following relations:

8 $$\frac{{R_{u2\% } }}{{H_{mo} }} = \gamma_{p} \left[ {1.65\xi_{m - 1,0} } \right]\;\;{\text{with}}\;{\text{a}}\;{\text{maximum}}\;{\text{of}}\;\;\frac{{R_{u2\% } }}{{H_{mo} }} = A\,\gamma_{p} \left[ {4 - \frac{1.5}{{\sqrt {\xi_{m - 1,0} } }}} \right]$$

where *H*_*mo*_ is the wave height, *γ*_*p*_ is the porosity coefficient, $\xi_{m - 1,0}$ is the Iribarren number.

In this study, the formulas derived by Van der Meer and Stam^8^, and Schimmels et al.^5^ are applied to determine the two percent wave run-up height.

### Group method of data handling

**Figure 2:**
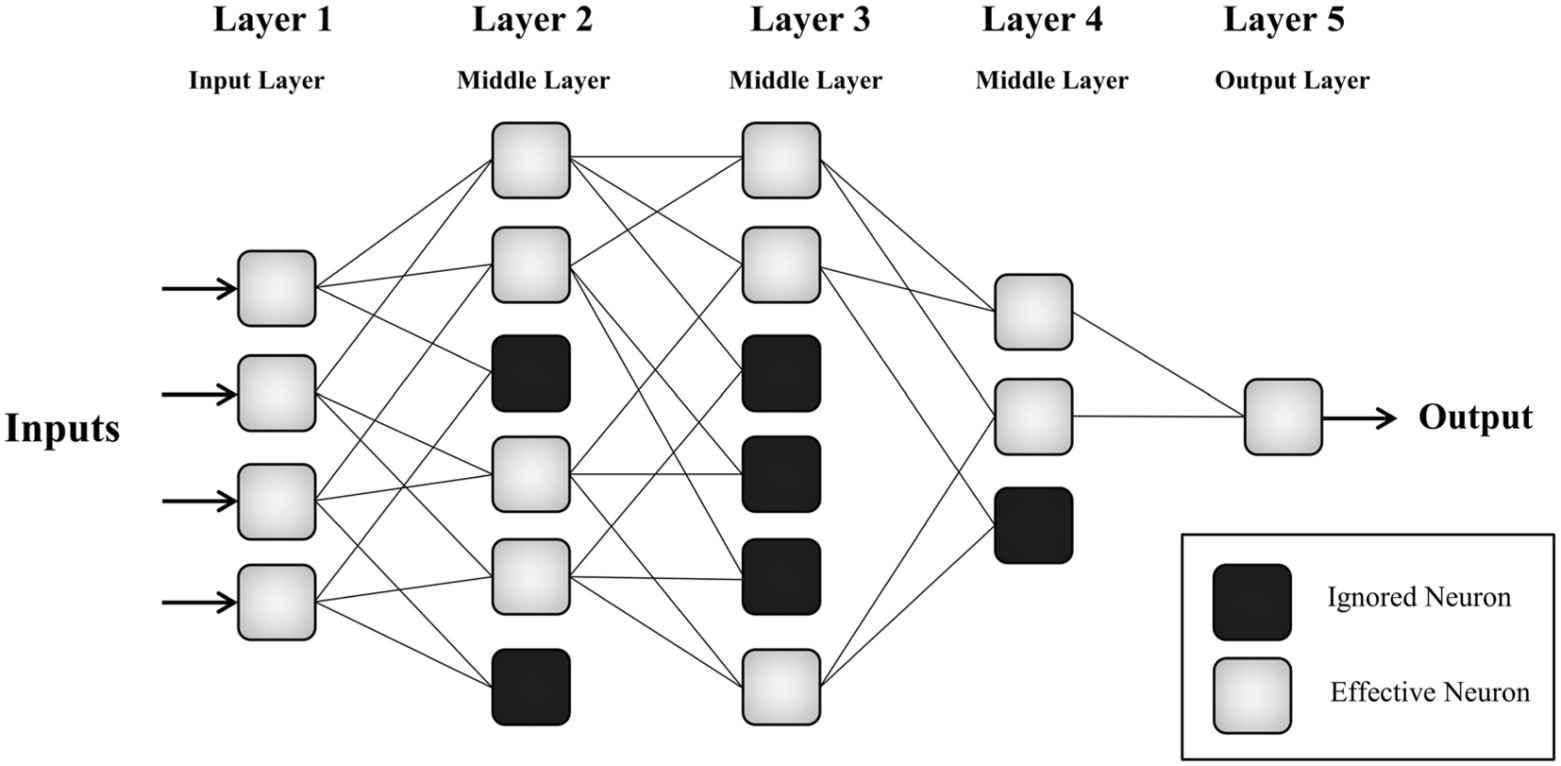
A schematic plot of GMDH structure with three hidden layers.

The group method of data handling (GMDH) is a machine learning model belonging to artificial neural networks (ANNs), which was introduced by Ivakhnenko^43^ for modeling complex systems. This method has been successfully applied in different fields of science and engineering. Similar to ANNs, the GMDH consists of neurons connected in different layers. In the GMDH network, the neurons in the next layer are produced as a combination of two neurons from the previous layer. Then, the output of each neuron is calculated by quadratic polynomial expressions, and the most effective neurons are selected to be connected to neurons in the next layer. In other words, the GMDH algorithm generates the structure of the network through successive generations of quadratic regression polynomials with two input variables or neurons. In Fig. 2, a schematic plan of a five-layer GMDH with effective and ignored neurons is shown.

The GMDH network is created and trained layer-by-layer and neuron-by-neuron. This strategy allows users to have access to the neurons’ information during the network training process. As can be seen in Fig. 2, in the second, third, and fourth layers, there are some ignored neurons. The ignoring of neurons and the selection of appropriate ones prevent the immense growth of the network. The number of ignored neurons in each middle layer is affected by the error evaluation criteria e.g. *MSE* criterion:

9 $$MSE = \frac{1}{N}\sum\limits_{i = 1}^{N} {(y_{i}^{m} - y_{i}^{o} )^{2} }$$

where *N* is the number of data, $y_{{}}^{p}$ is the predicted output of neurons in each layer, and $y_{{}}^{m}$ is the observed value. In the GMDH structure, contrary to conventional matrix structure, a number of mathematical equations are applied to speed up calculation process^44^. The output of each neuron is calculated according to Volterra–Kolmogorov–Gabor (VKG) polynomial. The second-order polynomial is incorporated in the structure of GMDH^43^ and is used in this study as the transfer functions in each neuron. The second-order polynomial may be written in the following form:

10 $$y = c_{0} + c_{1} x_{1} + c_{2} x_{2} + c_{3} x_{1}^{2} + c_{4} x_{2}^{2} + c_{5} x_{1} x_{2}$$

where *y* is the output, (*x*_*1*_, *x*_*2*_) is the input vector, and *c* is the weighting coefficient. The intricacy of the neurons will be increased layer by layer, which causes that the final network is becoming complex^67^. The weighting coefficients are calculated using regression techniques:

11 $$c = \left( {A^{T} A} \right)^{ - 1} A^{T} Y$$

where *c* represents the weighting coefficient vector, *A* denotes the following matrix:

12 $$A = \left[ \begin{gathered} 1\,\,\,\,\,x_{1}^{1} \,\,\,\,\,\,x_{2}^{1} \,\,\,\,\,x_{1}^{1} x_{2}^{1} \,\,\,\,\,\left( {x_{1}^{1} } \right)^{2} \,\,\,\,\,\,\left( {x_{2}^{1} } \right)^{2} \hfill \\ 1\,\,\,\,\,x_{1}^{2} \,\,\,\,\,x_{2}^{2} \,\,\,\,\,x_{1}^{2} x_{2}^{2} \,\,\,\,\,\left( {x_{1}^{2} } \right)^{2} \,\,\,\,\,\left( {x_{2}^{2} } \right)^{2} \hfill \\ \vdots \hfill \\ 1\,\,\,\,\,x_{1}^{m} \,\,\,\,x_{2}^{m} \,\,\,\,x_{1}^{m} x_{2}^{m} \,\,\,\,\left( {x_{1}^{m} } \right)^{2} \,\,\,\,\left( {x_{2}^{m} } \right)^{2} \hfill \\ \end{gathered} \right]$$

and *Y* is the matrix of outputs:

13 $$Y = \left\{ {y_{1} ,\,y_{2} ,\,\,...\,y_{m} } \right\}^{T}$$

in which *m* is the number of samples.

In this study, the number of layers is equal to 5. The maximum number of neurons in the next layer ($N_{np}^{i + 1}$) can be calculated by applying the following equation:

14 $$N_{np}^{i + 1} = \left( \begin{gathered} N_{np}^{i} \hfill \\ 2 \hfill \\ \end{gathered} \right) = \frac{{N_{np}^{i} !}}{{2! \times \left( {N_{np}^{i} - 2} \right)!}}$$

where $N_{np}^{i}$ is the number of neurons in the *i* layer. Substituting $N_{np}^{i}$ equal to eight i.e. the same as the number of input variables in Eq. (14), $N_{np}^{i + 1}$ results in 28. In this study, the maximum number of neurons in each layer was determined to be 10.

In summary, the following seven steps are taken to build the GMDH network (1) determining the GMDH structure-in this study 5 layers are considered for the network with a maximum of 10 neurons in each layer; (2) standardization of the data; (3) entering the data to the neurons of the next layer; (4) allocating the polynomial-based fit to each neuron in layers based on the values of two neurons of the previous layer; (5) calculating weights for a polynomial—Eq. 11; (6) calculating the output of the neurons and selected appropriateness of them—Eq. 9; (7) move to the next layer and repeat the steps of 3 to 6 to create the entire GMDH network.

### Hybrid technique

**Figure 3:**
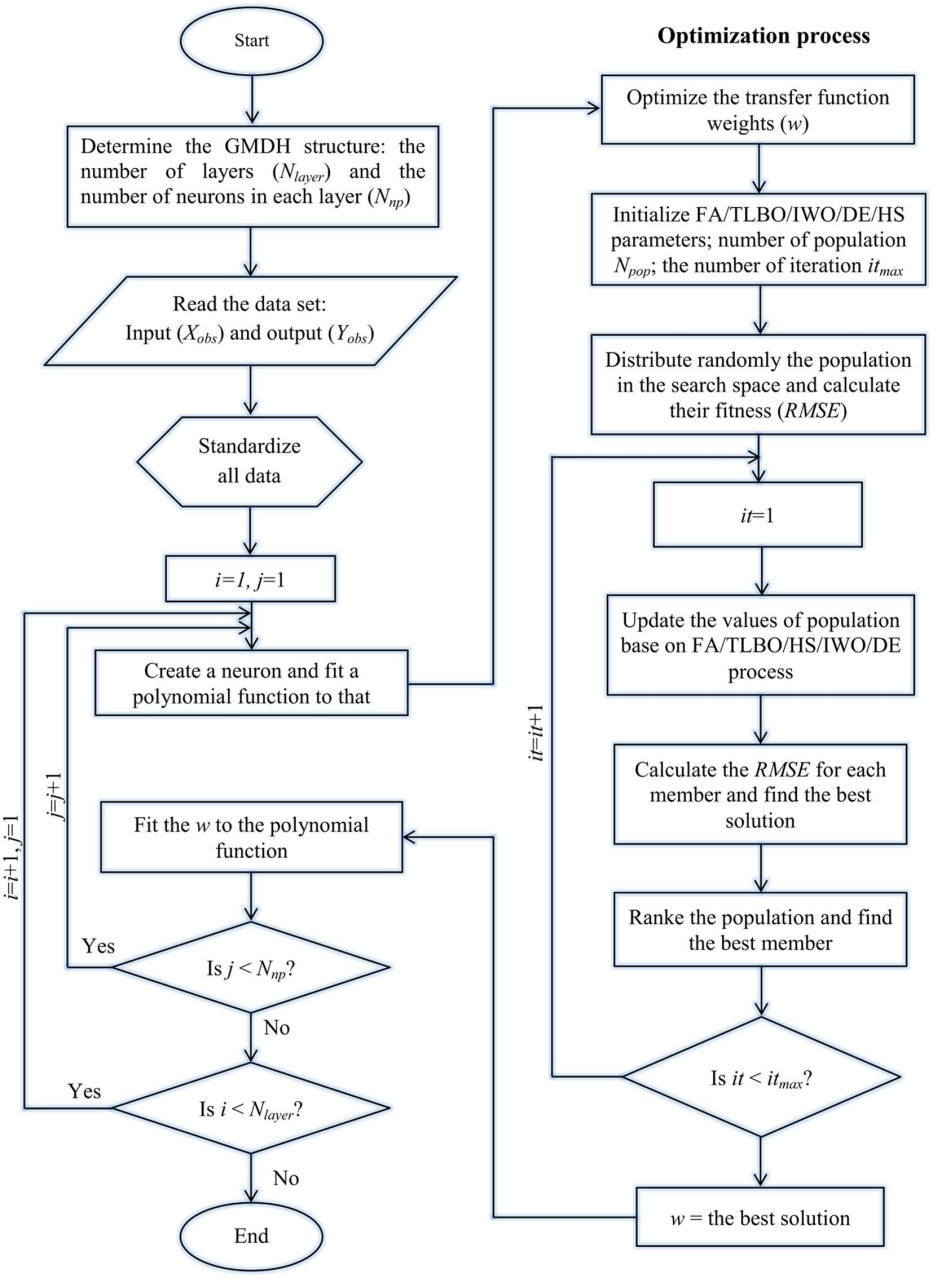
The general flowchart of the constructed hybrid GMDH models.

Instead of using the least-squares technique of GMDH, meta-heuristic optimization algorithms can be embedded with the GMDH model. This technique can be applied to optimize either the weighting coefficients in Eq. 10 or the structure of the network. In this study, meta-heuristic optimization algorithms are used to optimize the weighting coefficients. The main difference between the general execution procedure of the standard GMDH and hybrid GMDH is the calculation process of the fifth step of the GMDH network mentioned in the previous section. In hybrid GMDHs, the meta-heuristic optimization approaches will be employed to optimize the weights of the polynomial. At the first step, a number of candidate solutions are distributed in the search space. Each member of this population represents a solution to Eq. 10. The fitness of members is calculated by applying *RMSE*, and the population is ranked. At the next step, the new values of the members will be calculated by meta-heuristic algorithms. Henceforth, the fitness of the population will be calculated and the members will be ranked. This process will be repeated until the final iteration. In the end, the best member represents the optimized values of Eq. 10. In Fig. 3, the general flowchart for setting up the hybrid GMDH models is shown.

The applied algorithms comprise new swarm intelligent-based models including invasive weed optimization, IWO, firefly algorithm, FA, and teaching–learning-based optimization, TLBO, as well as evolutionary-based models including harmony search, HS, and differential evolution, DE. The integration of the GMDH model and IWO has been developed and executed for the first time in this study. In Table 1, descriptions of the applied algorithms are presented, and a brief description of these algorithms is provided in the following sections.

**Table 1:** Presentation of the meta-heuristic algorithms.

Category	Algorithms	Inspired by	Reason for the selection
Swarm intelligence	IWO	Plants and animals	High performance in combination with ANFIS and ANN models^45,46^
	FA	Insects movement	A robust and efficient algorithm in optimization problems^47,48^
	TLBO	Teaching–learning process	A simple and reliable algorithm that does not need any initial parameters^49,50^
Evolutionary	HS	Harmony in music	Successful application of hybrid GMDH-HS in modeling nonlinear problems^20,51^
	DE	Biological insights	Popular, simple, effective^52^ and successful applications of GMDH-DE^53^

### Invasive weed optimization

The invasive weed optimization (IWO) algorithm is inspired by weed colonization and was first proposed by Mehrabian and Lucas^54^. In this algorithm, a population of initial solutions (weeds) dispreads randomly in the entire search space. The fitness of weeds is evaluated and produces a number of seeds-the population with better fitness produces more seeds. Produced seeds are randomly distributed in search space by normally distributed random numbers with a mean equal to zero, but with a varying variance. The IWO applies the standard deviation (*σ*) of the random function which is defined between the ranges of the pre-defined initial value (*σ*_*initial*_) to a final value (*σ*_*final*_) and is calculated in each step from the following equation^54^:

15 $$\sigma_{iter} = \frac{{\left( {iter_{\max } - iter} \right)^{n} }}{{\left( {iter_{\max } } \right)^{n} }}\left( {\sigma_{initial} - \sigma_{final} } \right) + \sigma_{final}$$

where *σ *_*iter*_ is the standard deviation of the present iteration, *iter*_*max*_ is the maximum number of iterations, and *n* is the nonlinear modulation index usually set as 2^55^.

After some iterations, the number of produced plants reaches a maximum value. At this stage, competitive exclusion eliminates undesirable plants based on the fitness function. Consequently, those with better fitness would survive and are allowed to replicate. This process might be continued either reaching the maximum epoch or achieving the exact solution.

### Firefly algorithm

This algorithm was inspired by the flashing and illuminating behavior of the fireflies and was proposed by Yang^56^. This algorithm is based on three general rules, (1) all fireflies in the search space are considered to be unisex so they can be attracted to the others; (2) the attractiveness of a firefly is proportional to its light intensity. Hence, the brighter fireflies attract the less bright ones. In the case of fireflies of similar brightness, their movements are assumed to be random; (3) the brightness of a firefly is determined by the objective function. In FA, the new position of agents ($X_{i}^{t + 1}$) is calculated according to the following equation:

16 $$X_{i}^{t + 1} = X_{i}^{t} + \beta_{0} e^{{ - \gamma r_{ij}^{2} }} \left( {X_{j}^{t} - X_{i}^{t} } \right) + \alpha \varepsilon_{i}$$

where *a* is a randomized parameter (mutation coefficient), $\varepsilon_{i}$ is the random vector, *β*_*0*_ is the attractiveness at distance *r* = 0, and *γ* is the absorption coefficient of light. More information about the FA algorithm can be found in the original work of Yang^56^.

### Differential evolution

The differential evolution (DE) is a stochastic and population-based algorithm proposed by Storn and Price^57^ applied in global optimization problems. The main structure of DE is based on extracting individual differences from a current population to build a new population. In other words, it is one of the optimization algorithms which has both evolutionary and swarm intelligence features^58^. Mutation, crossover, and selection are evolutionary operators, while distance and direction of the population can be considered as swarm intelligence features.

Assuming three agents of the population as *X*_*i1*_, *X*_*i2*_, and *X*_*i3*_, the mutation scheme can be expressed as a trial vector ($v_{i}^{t}$) which will be developed for each member of the population by applying the following equation:

17 $$u_{i}^{t} = X_{i1}^{t} + F \times \left( {X_{i2}^{t} - X_{i3}^{t} } \right)$$

where *t* is the iteration index and *F* is the scale factor that controls the amount of differential variation.

In the crossover scheme, offspring ($u_{i1}^{t}$) will be generated as:

18 $$v_{ij}^{t} = \left\{ {\begin{array}{*{20}l} {u_{ij}^{t} } \hfill & {{\text{if}}\,{\text{rand}}_{i}^{j} (0,1) \in C_{r} \,\,\,{\text{or}}\,\,\,j = j_{i}^{{{\text{rand}}}} } \hfill \\ {X_{ij}^{t} } \hfill & {{\text{otherwise}}} \hfill \\ \end{array} } \right.$$

where *C*_*r*_ is the crossover controller assuming values between 0 to 1 and $j_{i}^{{{\text{rand}}}}$ is a random integer number between 1 and the dimension of the problem, *D*. In the end, the selection operator is applied and the new position is calculated from:

19 $$X_{i}^{t + 1} = \left\{ \begin{gathered} v_{i}^{t} \,\,\,\,\,\,{\text{if}}\,\,\,\,f(v_{i}^{t} ) \le f(X_{i}^{t} ) \hfill \\ X_{i}^{t} \,\,\,\,{\text{otherwise}} \hfill \\ \end{gathered} \right.$$

### Teaching–learning-based optimization

The teaching–learning-based optimization (TLBO) is inspired by the philosophy of the teaching and learning process and was proposed by Rao et al.^49^ The algorithm is based on the evaluation of the influence of a teacher on the performance of students. The TLBO consists of two main phases: Teaching and Learning Phases. Among all the designated populations in the search space, the best solution (base on fitness) is assigned to the teacher, and the learners would learn and update their knowledge from the teacher according to the teaching operation:

20 $$x_{i}^{new} = x_{i}^{old} + rand_{i} \times \left( {x_{best} - T_{F} \times x_{mean} } \right)$$

where $x_{i}^{new}$ is the new positions of the *i*th learner, $x_{i}^{old}$ is the old positions of the *i*th learner, *rand*_*i*_ is a random number between 0 to1*, x*_*best*_ is the position of the teacher, *x*_*mean*_ is the mean individual position of the current class, and *T*_*F*_ is the teaching factor that is applied to change the mean value.

After this stage, learners increase their knowledge by interacting randomly with other learners in class as the equation below:

21 $$x_{i}^{new} = \left\{ \begin{gathered} x_{i}^{old} = rand_{i} \times \left( {x_{i} - x_{j} } \right),\,\,if\,\,f\left( {x_{i} } \right) < f\left( {x_{j} } \right) \hfill \\ x_{i}^{old} = rand_{i} \times \left( {x_{j} - x_{i} } \right),\,\,\,otherwise \hfill \\ \end{gathered} \right.$$

where *x*_*j*_ is a member of the population that is selected randomly.

### Harmony search

The harmony search (HS) is a meta-heuristic optimization method inspired by musicians which simulate the improvisation process of the group of musicians^59^. To create the HS structure, some parameters, including the harmony memory consideration rate (*HMCR*), pitch adjusting rate (*PAR*), fret width (*FW*), and harmony memory size (HMS) have to be set at the initialization stage. The harmony memory is a matrix of candidate solutions. The *HMCR* varies between 0 and 1 and controls the balance between exploration and exploitation. The *PAR* and *FW* are useful parameters in adjusting the convergence rate of the algorithm. The quantity $X_{j} = \left[ {x_{1}^{j} ,x_{2}^{j} ,\,\,...\,\,,x_{D}^{j} } \right]$ represents the *j*th harmony vector. The HM is filled with the HMS harmony vectors:

22 $${\text{HM}} = \left[ \begin{gathered} x_{1}^{1} \,\,\,\,\,\,\,\,\,\,\,\,\,\,x_{2}^{1} \,\,\,\,\,\,\,......\,\,\,\,\,\,\,x_{D}^{1} \hfill \\ x_{1}^{2} \,\,\,\,\,\,\,\,\,\,\,\,\,x_{2}^{2} \,\,\,\,\,\,\,......\,\,\,\,\,\,\,x_{D}^{2} \hfill \\ x_{1}^{HMS} \,\,\,\,\,x_{2}^{HMS} \,\,\,\,......\,\,\,\,\,\,\,x_{D}^{HMS} \hfill \\ \end{gathered} \right]$$

At the first iteration, HM is filled with random solution vectors. In the next iterations, the HM must be updated with a new solution. If the new solution vector is better than the worst vector, then it is stored and replaces the worst vector.

### Experimental data and model setup

#### Datasets

**Figure 4:**
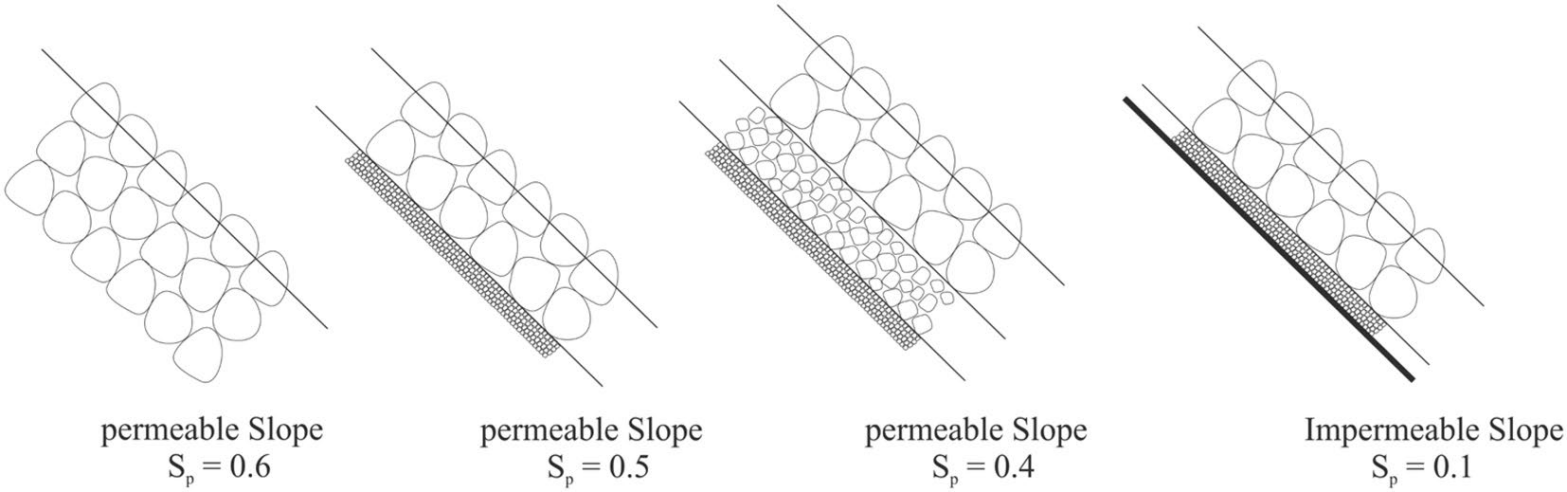
Bed permeability parameter for different types of bed materials.

In this study, eight parameters are used as the input vector to build and train the GMDH models for estimating the wave run-up (*R*_*u2%*_). The input vector includes spectral peak period (*T*_*P*_), mean wave period (*T*_*m*_), significant wave height (*H*_*s*_), beach slope (cot*α*), the relative size of bed material (*D*_*85*_*/D*_*15*_), the surf similarity parameter corresponding to the mean wave period ($\xi_{\text{m}}$), the surf similarity parameter corresponding to the peak period ($\xi_{p}$), and the bed permeability (*S*_*p*_). The *S*_*p*_ characterizes bed material especially particle grading and permeability. Figure 4 shows the range of *S*_*p*_ for different types of coastal slopes. The *S*_*p*_ data covers both impermeable and permeable slopes (see Table 2).

**Table 2:** Statistical summary of the data used in this study.

Parameter	Type	Data range	Average	Standard deviation (SD)	Correlation coefficient (*R*_*u2*%_)
*R*_*u2%*_ (m)	Output	[0.043,1.6]	0.237	0.211	1
*T*_*P*_ (s)	Input	[1.33,5.1]	2.542	0.884	0.351
*T*_*m*_ (s)	Input	[1.24,4.4]	2.181	0.707	0.403
*H*_*s*_ (m)	Input	[0.461,1.18]	0.139	0.138	0.938
cot*α*	Input	[1.5,4]	2.709	0.783	0.0003
*D*_*85*_*/D*_*15*_	Input	1.25,2.25	1.641	0.485	0.028
*S*_*p*_	Input	0.1,0.5,0.6	0.276	0.205	0.009
$\xi_{\text{m}}$	Input	[0.991,7.584]	3.202	1.367	0.0154
$\xi_{p}$	Input	[1.047,8.869]	3.740	1.681	0.0129

The 256 data set was extracted from Van der Meer and Stam^8^ report. All tests were conducted in a wave flume of 1 m wide, 1.2 m deep, and 50 m long. Data were measured for two types of particle grading including uniform rock and riprap, and different values of *S*_*p*_. In this study, all available parameters that have a direct effect on the wave run-up height were selected as input parameters for the predictive models. Information about the statistical properties of the dataset is presented in Table 2.

#### Evaluation criteria

In the present study, the statistical assessment of the training data set was conducted for checking the reliability of the developed GMDH models. A similar assessment of the testing data set was conducted for the evaluation of the performance of GMDH models and empirical relations. This was done by applying different statistical parameters including root mean square error (*RMSE*), determination coefficient (*R*^*2*^), mean absolute error (*MAE*), and the index of agreement (*IA*).

The *RMSE* determines the root mean square error between the observed and predicted values, while the *MAE* describes the distance between the observed and predicted values. The *R*^*2*^ indicates how well the predicted values fit the regression model. The *IA* is the measure of the agreement between the predicted and observed values. These parameters are calculated as follow:

23 $$RMSE = \sqrt {\frac{1}{N}\sum\limits_{i = 1}^{N} {(x_{i}^{m} - x_{i}^{o} )^{2} } }$$

24 $$MAE = \frac{1}{N}\sum\limits_{i = 1}^{N} {\left| {x_{i}^{m} - x_{i}^{o} } \right|}$$

25 $$R^{2} = \frac{{\left( {\sum\nolimits_{i = 1}^{N} {\left( {x_{i}^{m} - \overline{{x^{m} }} } \right)} \left( {x_{i}^{o} - \overline{{x^{o} }} } \right)} \right)^{2} }}{{\sum\nolimits_{i = 1}^{N} {\left( {x_{i}^{m} - \overline{{x^{m} }} } \right)^{2} \sum\nolimits_{i = 1}^{N} {\left( {x_{i}^{o} - \overline{{x^{o} }} } \right)^{2} } } }}$$

26 $$IA = \frac{{\sum\nolimits_{i = 1}^{N} {\left( {x_{i}^{m} - x_{i}^{o} } \right)^{2} } }}{{\sum\nolimits_{i = 1}^{N} {\left( {\left| {x_{i}^{m} - \overline{{x^{o} }} } \right| + \left| {x_{i}^{o} - \overline{{x^{o} }} } \right|} \right)^{2} } }}$$

where *N* is the number of data, $x_{i}^{m}$ denotes the predicted value, $x_{i}^{o}$ is the observed value, and bar stands for the average of the variables. The best fitting between models and observed data is for *RMSE* and *MAE* equal to 0, and *R*^*2*^ and *IA* equal to 1.

#### Models setup and structure

To begin with the model development process and implementation of data, the data set was divided randomly into a training set comprising 80% of the available data set and a testing set covering 20% of the data set. The general structure of the GMDH models is presented in Table 3. The allocated values of parameters are based on their values from the previous studies^60–64^. For all the developed models, 300 epochs were considered. It is worth noting that this iteration number was reached based on the convergence criteria of GMDH models.

**Table 3:** Structure of models and initial parameters of meta-heuristic algorithms.

Model	Structure		Meta-heuristic parameters		
	Number of middle layers	Maximum neurons in each layer	Parameter	Value	Epoch
GMDH	5	10	–	–	300
GMDH-IWO	5	10	Maximum no of seeds	10	300
			Minimum no of seeds	1	
			Initial Standard deviation (*σ*_*initial*_)	0.5	
			Final Standard deviation (*σ *_*final*_)	0.001	
			Search Space Range	[−10,10]	
GMDH-FA	5	10	Mutation Coefficient (*a*)	0.2	300
			Attraction Coefficient (*β*_*0*_)	2	
			Light Absorption Coefficient (*γ*)	1	
			Search Space Range	[−10,10]	
GMDH-DE	5	10	Lower bound of scaling factor	0.2	300
			Upper bound of scaling factor	0.8	
			Crossover controller (*C*_*r*_)	0.9	
			Search space range	[−10,10]	
GMDH-HS	5	10	Fret width damp ratio	0.995	300
			Pitch adjustment rate (*PAR*)	0.1	
			Harmony memory consideration rate (*HMCR*)	0.9	
			Search space range	[−10,10]	
GMDH-TLBO*	5	10	Search space range	[−10,10]	300

*The TLBO algorithm can be applied without allocating any specific primary or adjusting parameter.

## Results

In this study, the GMDH model and the five hybrid GMDH models described in the previous section were used to estimate the wave run-up. Results obtained for the training stage are presented in Table 4.

**Table 4:** Statistical parameters for the evaluation of the performance of derived models at a training stage.

Method	Statistics			
	*RMSE* (m)	*R*^*2*^	*MAE* (m)	*IA*
GMDH-TLBO	0.0250	0.9863	0.0184	0.9965
GMDH	0.0254	0.9853	0.0195	0.9963
GMDH-DE	0.0274	0.9829	0.0209	0.9956
GMDH-FA	0.028	0.9823	0.0188	0.9955
GMDH-IWO	0.0355	0.9759	0.0198	0.9932
GMDH-HS	0.0359	0.9730	0.0219	0.9928

The results in Table 4 show that the applied GMDH models are capable of modeling wave run-up, which confirm low values of *RMSE* and *MAE,* and the close to one values of *R*^*2*^ and *IA*. Among all methods developed in this study GMDH-TLBO, with the lowest *RMSE* and *MAE* (*RMSE* = 0.0254 m and *MAE* = 0.0195 m) and the highest *R*^*2*^ and *IA* (*R*^*2*^ = 0.9863 and *IA* = 0.9965) provide the best results at the training stage. However, the results show that HS, FA, DE, and IWO, decrease the performance of the standard GMDH. Nevertheless, the decision on the superiority of the derived models can be conducted by the evaluation of the results at the testing stage. In this respect, Table 5 summarizes the **s**tatistical parameters derived for the evaluation of the performance of developed models at a testing stage.

**Table 5:** **S**tatistical parameters for the evaluation of the performance of derived models at a testing stage.

Method	Statistics				Enhancement ( +) or deterioration (−) of the *RMSE* in performance (%)	Establishment	Execution time
	*RMSE* (m)	*R*^*2*^	*MAE* (m)	*IA*			
GMDH-FA	0.0209	0.9908	0.0172	0.9977	+ 47.49	Difficult	High
GMDH-IWO	0.0220	0.9908	0.0170	0.9975	+ 44.74	Difficult	High
GMDH-TLBO	0.0235	0.9888	0.0180	0.997	+ 41.08	Difficult	High
GMDH-HS	0.0262	0.9864	0.0211	0.9964	+ 34.26	Difficult	High
GMDH-DE	0.0302	0.984	0.0240	0.9953	+ 24.12	Difficult	Medium
GMDH	0.0398	0.9674	0.028	0.9916	Base	Not Simple	Low
Schimmels et al.^5^	0.0763	0.9549	0.0638	0.9711	−91.52	Simple	Low
Van der Meer and Stam^8^	0.1224	0.8178	0.0747	0.927	−207.22	Simple	Low

**Figure 5:**
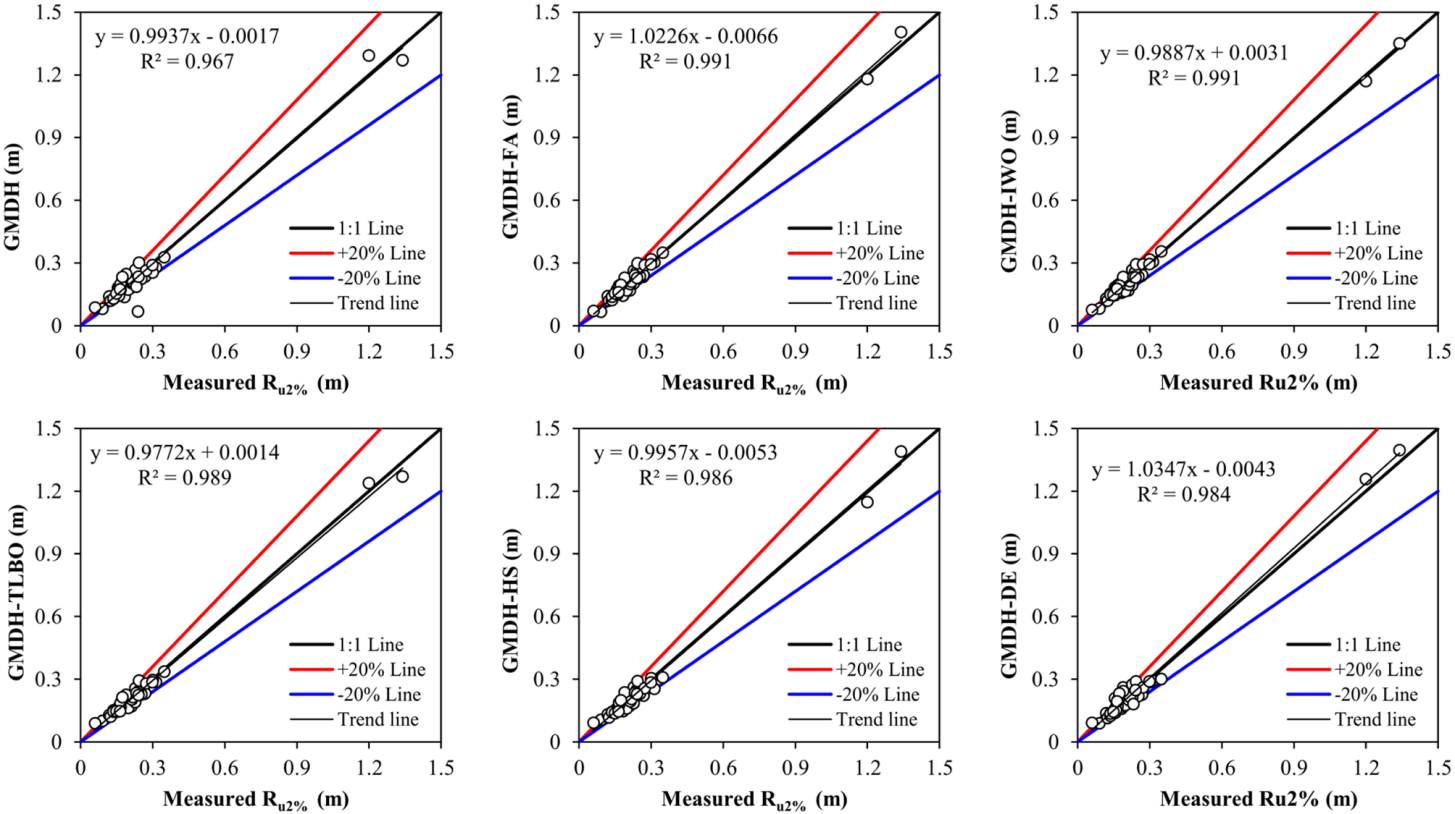
Scatter plots obtained for the testing stage.

The results in Table 5 show that the standard and hybrid GMDH models provide more accurate results than the empirical relations. However, the development of the derived models is a time consuming process and their execution requires more time in comparison to the empirical models. The average values of *RMSE*, *MAE*, *R*^*2*^, and *IA* for the GMDH models are 0.027 m, 0.0209 m, 0.9847, and 0.9959, respectively, whereas for empirical relations are 0.0993 m, 0.0692 m, 0.886, and 0.9490, respectively. The developed GMDH models improve the prediction of wave run-up by about 72.81% in comparison to the empirical methods. Among the developed GMDH models, the GMDH-FA with the lowest values of *RMSE* and *MAE* (*RMSE* = 0.0209, *MAE* = 0.0172) and the highest *R*^*2*^ and *IA* (*R*^*2*^ = 0.9908, *IA* = 0.9977) can be considered as the most precise predictive model. The application of meta-heuristic optimization algorithms improves the performance of the standard GMDH model by 39.70%. The performance of most hybrid GMDH models is lower at the training stage than the standard GMDH. The results confirm a relatively good performance of the derived models at the testing stage. The standard GMDH model may trap into the local solutions, which causes the over-fitting problem. Figure 5 presents the scatter plots for the testing stage. Scatter points of the hybrid GMDH models are closer to 1:1 line than corresponding points obtained by applying the standard GMDH technique, which indicates that the meta-heuristic algorithms increase the performance of GMDH.

**Figure 6:**
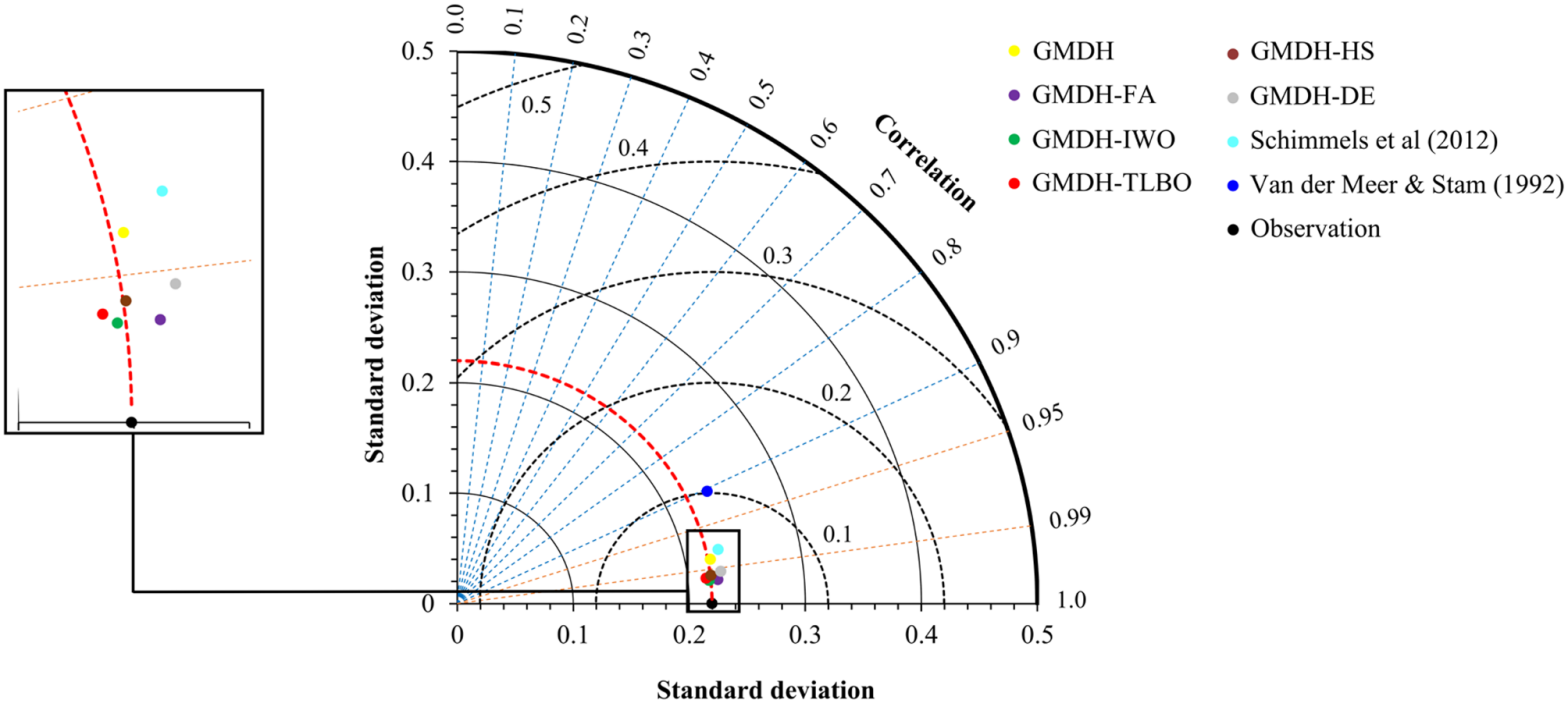
Taylor diagram for the tested data set.

To provide further insight into the outcomes of the derived models, the results of the models are plotted in the Taylor diagram^65^. The purpose of the Taylor diagram is to present on a single plot three statistical indices including the standard deviation, centered *RMSE,* and correlation. In Fig. 6, the values on the vertical and horizontal axes represent the standard deviation, the values on the dashed lines represent the correlation, and the values on the dash-curved lines represent the centered *RMSE*. Figure 6 shows that the dots representing the GMDH models are closer to the observation points than corresponding dots obtained by the application of the empirical relations. The results of the standard and hybrid GMDH models are magnified in a separate box. The plots show the superiority of the GMDH-FA over the remaining GMDH models.

### Further discussion

An important factor in the analysis of the performance of machine learning methods and meta-heuristic techniques is the speed of the derived algorithms. Based on the observed CPU time presented in Table 6, it is becoming clear that the standard GMDH model is more efficient in terms of computational cost than the derived hybrid GMDH models. It is worth mentioning that the application of empirical equations in practical projects is easier than the ML models. However, nowadays, with tremendous progress that has been made in technology and minicomputers, the applications of ML- trained models to determine wave run-up, is straightforward.

**Table 6:** The CPU time for 300 epochs of the applied GMDH models.

Model	Empirical relations and standard GMDH	GMDH-IWO	GMDH-FA	GMDH-DE	GMDH-HS	GMDH-TLBO
CPU* time (min)	< 1	116	405	11	43	116

*Core i7; RAM: 8 GB.

In the previous section, the analyses of the performance of the GMDH models versus the empirical relations showed the superiority of the developed GMDH models. But in spate of that, the question may arise whether the differences between the results obtained by applying the developed models are statistically significant. Thus, the Kruskal–Wallis tests were carried out and the results are presented in Table 7. The non-parametric Kruskal–Wallis test is a technique often applied in statistical analyses. Mahdavi-Meymand et al.^18^ used the Kruskal–Wallis test to compare several machine learning (ML) techniques and empirical equations applied to predict spillways air demand and reported that there is no significant difference at the 99% confidence level between the applied ML approaches.

**Table 7:** The results of the Kruskal–Wallis test for assessing the significant statistical differences between the applied models.

Methods	*p*-value	Significantly different (95%)	Significantly different (99%)
Schimmels et al.^5^, Van der Meer and Stam^8^	0.7251	NO	NO
GMDH, GMDH-FA, GMDH-DE, GMDH-IWO, GMDH-TLBO, GMDH-HS, Schimmels et al.^5^, Van der Meer and Stam^8^	˂.0006	YES	YES
GMDH, GMDH-FA, GMDH-DE, GMDH-IWO, GMDH-TLBO, GMDH-HS	0.994	NO	NO

The results in Table 7 show that the probability value of the Kruskal–Wallis test for the empirical relations is 0.7251, which is higher than 0.05 and 0.01. This shows that there are no significant statistical differences between the results obtained by applying two empirical relations at both 95% and 99% confidence levels. However, significant differences exist between the results obtained by applying the GMDH models and empirical relations (*p*-value ˂0.0006). Thus, the GMDH models may be recommended to be applied instead of empirical relations to predict wave run-up. Moreover, although the statistical criteria confirmed that meta-heuristic algorithms increase the efficiency of the GMDH, the results in Table 7 show that there is no significant statistical difference between the outcome of different GMDH models. Hence, it is recommended to use the standard GMDH in situations where computational time is an important factor for users. More insight into the results obtained in the modelling of wave run-up in coastal regions is provided in Table 8.

**Table 8:** Summary of the studies conducted to predict wave run-up in coastal regions.

Authors	Methods	Type of the machine learning model	Calculated *R*^*2*^	Remarks
Abolfathi et al.^12^	M5’ Decision tree	Decision tree	0.970	The results show the general ability of M5’ to simulate wave run-up
Bakhtyar et al.^66^	ANFIS	Hybrid intelligent systems	0.960	The comparison of results confirms the high accuracy of ANFIS in predicting wave run-up
	Empirical formulas	–	0.890	
Erdik and Savci^7^	ANFIS	Hybrid intelligent systems	0.621	TS Fuzzy is a capable tool for modeling wave run-up
	Empirical formulas	–	0.559	
Bonakdar and Etemad-Shahidi^10^	M5 model tree	Decision tree	0.920	M5 results are better than TS Fuzzy and empirical formulas
	TS Fuzzy	Hybrid intelligent systems	–	
	Empirical formulas	–	0.902	
Elbisy^11^	MART*	Decision tree	0.974	The MART model is more accurate than the ANN
	ANN	Neural computing	0.837	
Rehman et al.^18^	ANN	Neural computing	0.995	Both ANN and RSM are robust methods for the prediction of wave run-up
	Response surface methodology (RSM)	–	0.999	
Yao et al.^19^	ANN	Neural computing	0.987	The ANN performance in predicting wave run-up was confirmed
The current study	GMDH	Neural computing	0.985	The results show that the GMDH models provide more accurate results than empirical relations. Applications of optimization algorithms increases the accuracy of standard models. The Kruskal–Wallis test shows that there are no significant statistical differences between the classical and hybrid GMDH models
	GMDH-TLBO GMDH-FA GMDH-DE GMDH-HS GMDH-IWO	Hybrid intelligent systems	0.986 0.991 0.984 0.973 0.976	
	Van der Meer and Stam^8^ Schimmels et al.^5^	–	0.818 0.955	

*Multiple additive regression trees.

Based on the findings of this study following potential subjects are proposed for future studies:

- Due to literature restrictions, it is recommended to take into consideration other possible future data resources to further evaluate predictive models developed in the present study.
- There is not a robust formula or procedure to select the best architecture of the GMDH model. More studies should be conducted to facilitate the construction of GMDH models.
- Based on the data used in this study and related calculated values of the correlation coefficient between the input and output variables, it was found that the beach slope has a limited effect on wave run-up height. Future laboratory studies should take into account a wider range of beach slopes.
- It is recommended to consider other non-parametric statistical tests such as Mann–Whitney test and evaluate the results of different methods.

## Conclusion

The ability to accurately estimate the maximum wave run-up is vital for the maintenance and development of coastal areas and the safety of the coastal zone population. In this study, hybrid swarm and evolutionary intelligent GMDH models as well as the standard GMDH technique were developed and applied to predict the two percent value of the wave run-up (*R*_*2*%_). The invasive weed optimization (IWO), firefly algorithm (FA), differential evolution (DE), teaching–learning-based optimization (TLBO), and harmony search (HS) optimization algorithms were used as the meta-heuristic optimization methods to train the GMDH. The results show that the GMDH models have better performance than the empirical relations. The Kruskal–Wallis tests show significant statistical differences between the results of the empirical relations and GMDH models.

The results show that the application of meta-heuristic optimization algorithms improves the performance of the standard GMDH model by 39.70% at the testing stage. However, the performance of most hybrid GMDH models is lower than the standard GMDH at the training stage. Among all developed models, the GMDH-FA provides the best results in the testing stage with *RMSE* = 0.0209 m and *IA* = 0.9977. Moreover, the results show that among all methods developed in this study GMDH-TLBO, with the lowest *RMSE* = 0.0254 m and *MAE* = 0.0195 m, and the highest *R*^*2*^ = 0.9863 and *IA* = 0.9965.

The computational costs indicate that the standard GMDH model and empirical equations are significantly faster than the embedded meta-heuristic GMDH techniques. Moreover, the results show that there are no significant statistical differences between the GMDH models and meta-heuristic algorithms. Hence, the application of time-consuming models such as GMDH-FA is not recommended in situations where computational cost is a decisive factor.
